# Erythrocyte binding activity of *Plasmodium vivax* tryptophan rich antigens is inhibited by patients’ antibodies

**DOI:** 10.1186/1475-2875-11-S1-P98

**Published:** 2012-10-15

**Authors:** Rupesh K Tyagi, Yagya D Sharma

**Affiliations:** 1 Department of Biotechnology, All India Institute of Medical Sciences, Ansari Nagar, New Delhi 110029, India

## Background

*Plasmodium vivax* uses duffy antigen to invade the human erythrocytes but reports of duffy negative infection by *P. vivax* indicate that this parasite may also be using some other alternative receptors for the invasion [1]. Studies are therefore required to identify these additional parasite molecules which are involved in host-parasite interaction. *Plasmodium vivax*, contains a family of tryptophan rich antigens containing positionally conserved tryptophan residues. These antigens can induced significant cellular and humoral responses in human individuals [2,3] and few showed interaction with erythrocytes.

## Materials and methods

Recombinant *Plasmodium vivax* tryptophan-rich antigens were purified by Ni-NTA affinity chromatography. Heparinized blood was collected from healthy lab individuals to obtain human erythrocytes for binding assays, and from the microscopically confirmed *P.vivax* infected individuals for patients’ sera. Rabbit antibodies against PvTRAgs used here were raised in the lab. Erythrocyte binding assays were performed by cell ELISA and Flow cytometry [2,4].

## Results

PvTRAgs binds to the human erythrocytes in a concentration dependent manner and not to the human lymphocytes. The binding was specific as competition with untagged proteins inhibits the erythrocyte binding to 50% at equimolar concentration. Antibodies raised against rabbits and produced by infected patients inhibit the binding of respective PvTRAg at different dilutions. PvTRAg38 and PvATRAg74 was sensitive to chymotrypsin only and not to the trypsin and neuraminidase while all other PvTRAgs were resistant to all these proteases.

## Conclusions

Six out of fifteen PvTRAgs bind specifically to the human erythrocytes and were inhibited by rabbit anti PvTRAgs and also by the antibodies produced during natural course of *P. vivax* infection. There may be more than one RBC receptor for these six PvTRAgs where only PvTRAg38 and PvATRAg74 are sensitive to chymorypsin while others using those molecules which are other than glycophorin and sialoglycoproteins. Studies are in progress to investigate the respective RBC receptors for these PvTRAgs and their role in erythrocyte invasion process.
